# XVII International AIDS Conference: From Evidence to Action - Clinical and biomedical prevention science

**DOI:** 10.1186/1758-2652-12-S1-S4

**Published:** 2009-10-06

**Authors:** Mark Mascolini, Rodney Kort, David Gilden

**Affiliations:** 1 Mark Mascolini, Allentown, 18102, USA; 2 Kort Consulting, Toronto, M4Y 2T6, Canada; 3 David Gilden, New York, 10025, USA

## Abstract

The question of whether to initiate ART at higher CD4+ cell counts than currently recommended by World Health Organization (WHO) treatment guidelines received much attention at the XVII International AIDS Conference (AIDS 2008). If studies presented at the conference ultimately lead to a revision of WHO treatment guidance, the estimated number of people who will need ART globally will increase substantially. Task-shifting is emerging as an important strategy for dealing with the acute shortage of health care workers in many high-burden countries, and several studies presented at AIDS 2008 demonstrated the impressive health system efficiencies garnered by using nurses or other health care providers to deliver HIV care and treatment. Other key presentations and discussion at the conference focused on the optimal time to start TB treatment in HIV-infected patients, the growing risk of resistance in high-burden countries, including its impact on future treatment options, and several large cohort trials testing optimal drug regimens in resource-limited settings.

Biomedical prevention research continues to confirm the long-term, protective benefits of circumcision. Several studies involving HIV serodiscordant heterosexual couples have produced data suggesting a strong protective effect of ART for HIV-negative partners. Disappointing results from recent vaccine and non-ARV based microbicides trials are nevertheless providing important data to this field, and the expanding number of pre-exposure prophylaxis (PrEP) trials and ARV-based microbicides appear to provide the best hope for a new, efficacious biomedical prevention intervention.

## Discussion

AIDS 2008, of course, took place two years before the deadline for universal access, and significant attention was devoted to the theme of the conference: Universal Action Now!, particularly in the context of both increases in the pace of scaling up treatment and care interventions and the growing realization that few countries are on target to meet universal access goals. Strategies for improving access to care in resource-limited countries, the risk of antiretroviral resistance in these countries, and evolving antiretroviral tactics dominated the AIDS 2008 agenda on clinical research and treatment of HIV infection.

### Should ART start at a higher CD4 count?

As AIDS 2008 began, an international panel of treatment experts convened by the IAS-USA updated antiretroviral treatment guidelines for adults. The panel recommended broadening options for starting ART at a CD4 count above 350 cells/mm^3 ^and to include people with active hepatitis B or C infection, cardiovascular disease risk, or compromised kidney function [1] The panel set no upper CD4 limit on when treatment should begin. A growing data stream from recent trials suggests that earlier ART may ward off not only AIDS-defining diseases, but also non-AIDS cancers and heart, liver, or kidney diseases [2-4].

A cohort study at AIDS 2008 added to accumulating evidence favouring earlier ART. This 1,679-person analysis of the US HIV Outpatient Study cohort found that a CD4 count under 350 cells/mm^3 ^when first measured independently raised the risk of new cardiovascular disease more than 75% [5] Additional evidence supporting the clinical value of earlier intervention with antiretrovirals could lead World Health Organization (WHO) advisors to review guidelines on when to start ART in resource-limited countries. WHO currently recommends ART for anyone with a CD4 count below 200 cells/mm^3^, while suggesting clinicians should "consider treatment" for people with 200 to 350 cells/mm^3 ^and defer treatment for people with more than 350 cells/mm^3 ^[6].

During AIDS 2008 incoming IAS President Julio Montaner predicted that revamped treatment guidelines for high-income countries could "revolutionize the treatment of HIV" by recognizing HIV infection as a chronic inflammatory disease that "affects the heart, liver, kidneys, and in due course we are going to learn the rest of the assorted organs in the body" [7,8]. Montaner cautioned that raising the CD4-cell threshold for starting ART could further widen the treatment-access gap between developed and developing countries unless experience confirms his modeling study, which suggests that expanding ART access will help limit the growth of the HIV epidemic and its associated costs by reducing infectivity. If this hypothesis proves true, the preventive effect of ART will be a powerful new argument for rolling out antiretroviral therapy more aggressively [9].

Related to the growing debate regarding optimal start and switch times is the issue of how and what information to use in clinical decision-making regarding switching drug regimens. A Haitian study raised concerns about relying on clinical or immunological criteria to detect ART failure, based on WHO treatment guidelines, in the absence of VL and CD4+ laboratory monitoring. In this study almost half (47%) the participants had HIV RNA levels below the limit of detection after being assessed as failing ART using clinical criteria, suggesting that the lack of VL PCR and other laboratory diagnostics in clinical decision-making could lead to premature switching [10].

#### When to start ART in TB co-infected patients

In many parts of the world, tuberculosis is the first AIDS diagnosis and a leading cause of death among people living with HIV. Yet the best time to start ART in HIV/TB-co-infected individuals remains controversial. Two studies presented at AIDS 2008 - one in Brazil and one in Argentina - addressed this question but did not reach the same conclusion.

**Figure 1:**
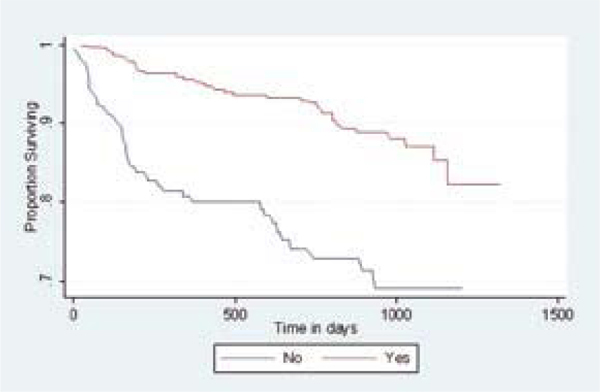
**Kaplan-Meier: survival after a TB diagnosis, by exposure to HAART (Log-rank test-p < .001)**. Source: Saraceni, V et al. Tuberculosis, HAART use and survival in the THRio Cohort, Rio de Janeiro, Brazil. (MOAB0305)

Valéria Saraceni's 632-person analysis of THRio, a Brazilian observational cohort study, found that starting ART *at any point *after beginning anti-TB therapy independently halved the risk of death, while completing the course of anti-TB drugs independently lowered the risk of death more than 85% [11] (Figure 1). A study of 142 HIV/TB co-infected people in Argentina recorded a higher overall death rate in those beginning ART within 8 weeks of starting anti-TB medications compared with those starting ART later (14.4% versus 6.8%, P = 0.013) [12]. However, TB-related mortality was the same in the two groups, a pre-ART clinical AIDS diagnosis was twice as common in the early-ART group, and the investigators did not perform multivariate analyses to determine whether the timing of ART affected mortality independent of other risk factors. Both of these studies were observational; ongoing randomized trials to address the optimal time to start ART during TB treatment are ongoing.

The Brazilian study linked delayed HIV diagnosis in TB patients with a lower chance of receiving ART. That finding underscores the critical need to integrate HIV and TB care, a policy emphasized in the WHO/IAS/Global Fund/World Bank consensus statement on knowledge gaps in the public health approach to delivering ART and care [13]. The Brazilian investigators recommended universal opt-out HIV testing for everyone with TB. UNAIDS already recommends HIV testing and counselling for all TB patients and screening of all HIV-infected people for TB. Because TB is such an important co-morbidity throughout the world, more rigorous prospective research is needed to optimize the role of ART for co-infected people.

In a post-conference development related to this issue, the South African chief director of HIV and AIDS, Dr Nomonde Xundu, confirmed that, as a result of evidence presented at the conference, a recommendation was under discussion about potential changes to South Africa's national treatment guidelines, including whether to recommend earlier initiation of ART, and whether additional clinical guidance for individuals co-infected with TB is also required [14].

#### Task shifting to widen access to care and treatment

Acute shortages of health professionals remain a stumbling block to wider HIV care in many low-income countries. New research presented by several groups at AIDS 2008 examined the role of task shifting - transferring certain physician responsibilities to other health workers - as a way to improve overall access to care.

**Figure 2:**
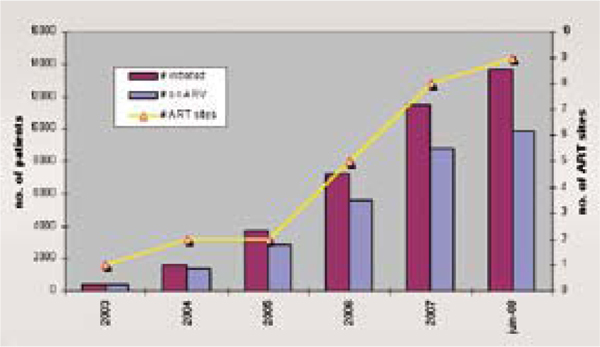
**ART initiation in Thyolo district 2003-2008**. Source: Massaquoi, M et al. Achieving universal access to antiretroviral therapy in a rural district in Malawi: how was it done? (TUAB0303).

In Malawi's rural Thyolo district, shifting some counselling work from nurses to lay counselors - then shifting ARV initiation duties from physicians to nurses - helped the region reach universal access targets [15]. The region has 600,000 people with HIV infection, including 9,000 to 12,000 who urgently needed antiretrovirals, when task shifting began. Médecins Sans Frontières set an initial target of treating 10,000 people. Shifting antiretroviral care duties away from physicians more than doubled the number of people tested for HIV, and, from 2004 through 2007, boosted the number of people starting ART from 2,000 to 12,000 (Figure 2). Universal access in Thyolo cost 3 Euros per district inhabitant per year.

**Figure 3:**
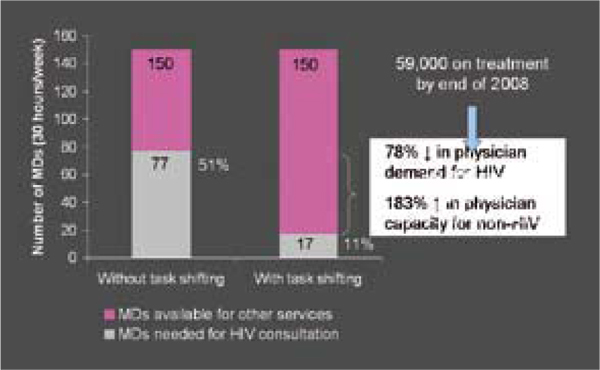
**Roll Up to national level**. Source: Chung, J et al. Quantification of physician-time saved in a task shifting pilot programme in Rwanda. (WEAB0205)

A study comparing nurse-led primary care-based ART with specialist hospital-based care in rural Swaziland documented lower mortality in the nurse-led setting and comparable dropout rates. A separate modeling study predicting the impact of a pilot task-shifting project in Rwanda estimated that the number of physicians needed to provide ART by the end of 2008 will drop from 77 physicians working 30 hours per week to 17 physicians working the same number of hours. That change represents a 78% decline in physician demand for HIV care and a 183% gain in physician capacity for non-HIV care (Figure 3).

Task shifting to nurses will not solve ARV access problems in sub-Saharan Africa because the region also suffers from a servere shortage of nurses. However, as the study in Malawi found, certain nursing tasks can be assigned to other workers, and greater community involvement can also expand HIV care [16]. By showing that task shifting may free physicians to manage other diseases, the Rwandan study demonstrates that robust AIDS funding need not mean neglect of other pressing clinical issues [17].

#### Risk of resistance in high-prevalence countries

With respect to ART, one of the most important AIDS 2008 studies assessed the emergence of resistance-related mutations in Malawi sites that rely on CD4 counts and clinical symptoms to assess treatment response - because routine viral load monitoring remains too expensive [18]. Resistance testing of samples from 96 people whose first ARVs failed uncovered an array of mutations that could severely compromise nucleoside use in second-line regimens.

Viral load monitoring would have detected ARV failure earlier and prevented the emergence of many mutations that developed while these patients continued a failing regimen. But viral load testing - and often CD4-cell assays - remain rare in many resource-poor clinics, and their absence limits optimal ART. Second-line antiretrovirals are more scarce than first-line agents in many of these same regions, and rampant resistance will threaten their use [19]. A recent modeling study by A Phillips (Royal Free and University College Medical School, United Kingdom) suggested that tracking symptoms and CD4 counts may do as well as viral load testing in increasing potential life-years survived in low-income countries [20]. But that analysis may have underestimated the impact of certain mutations detected in the Malawi study.

A 2008 consensus statement by WHO, IAS, World Bank and the Global Fund underscored the need to prioritize research to address two concerns raised by this study - determining the optimal time and criteria for switching to second-line therapy, and defining the most appropriate use of viral load and CD4-cell monitoring in resource-constrained regions [21]. The answers to these questions will be key in shaping the "second wave" of ART rollout and the clinical approach to treatment and care in low- and middle-income countries.

#### Trials of preferred ARV regimens

Many randomized ARV trials recruit patients from across the globe. Often, however, these trials enrol patients from the same well-established clinics in low- and middle-income countries. Rigorously testing promising ARV tactics in diverse settings is critical to establishing their value in countries with differing demographics and standards of care. Two ongoing trials tackling this issue were presented at AIDS 2008.

**Figure 4:**
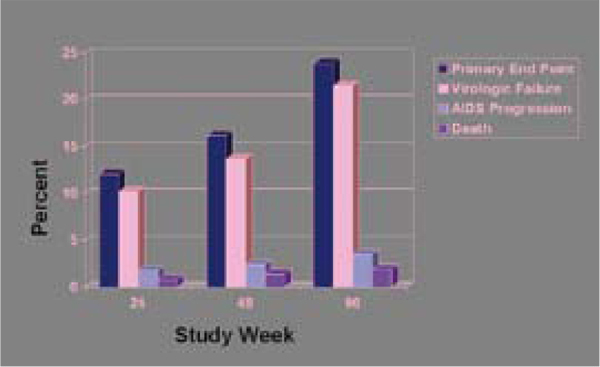
**Cumulative probability of treatment failure ddI-EC+FTC+ATV**. Source: Campbell, T et al. ACTG A5175: a multinational study of ddI-EC, FTC and atazanavir vs. co-formulated AZT/3TC and efavirenz for initial treatment of HIV-1 infection. (THAB0404)

The multinational PEARLS trial led by the US AIDS Clinical Trials Group set out to compare three first-line regimens in 1,361 adults in Brazil, Haiti, Peru, Malawi, South Africa, Zimbabwe, India, and Thailand, and 210 adults in the United States [22]. Patients randomized to once-daily didanosine, emtricitabine, and atazanavir (without a ritonavir boost) had a higher risk of treatment failure after 72 weeks than patients randomized to twice-daily zidovudine plus lamivudine and once-daily efavirenz (Figure 4). Failure rates differed from country to country and were higher among people with prior or current tuberculosis. Two points stand out: First, the atazanavir regimen failed by viral load criteria before clinical failure criteria became statistically significant - a finding emphasizing the value of viral load monitoring. Second, although atazanavir is licensed for use with or without ritonavir, most patients in the US and Western Europe take the drug with ritonavir to keep atazanavir concentrations even.

**Figure 5:**
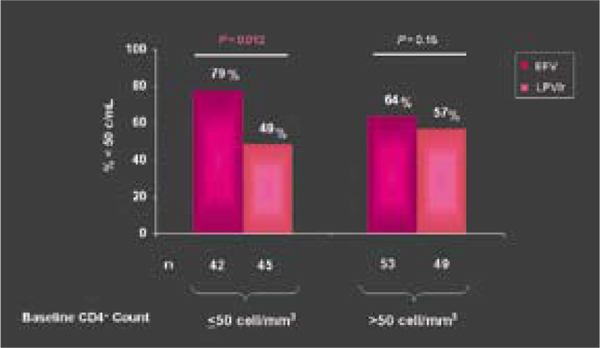
**Virological suppression sratified by baseline CD4+ counts (>/< 50 cell/mm^3^)**. Source: Madero, S. et al. A prospective, randomized, open label trial of efavirenz versus lopinavir/ritonavir based HAART among antiretroviral therapy naïve, HIV infected individuals presenting for care with CD4 cell counts. (TUAB0104)

In a recent US trial published before the conference an efavirenz-based combination controlled HIV better than lopinavir/ritonavir in previously untreated people, even in those starting ART with a viral load above 100,000 copies/mL [23]. A 48-week Mexican trial led by independent investigators confirmed better viral control with efavirenz than with lopinavir/ritonavir in ARV-naive people with advanced HIV infection (Figure 5) [24]. The Mexican study enrolled only patients with fewer than 200 CD4 cells/mm^3^, and the median pre-treatment count stood well under 100 cells/mm^3^. Discovery of compelling country-specific results in such studies should inspire further randomized controlled trials outside high-income countries.

#### Analyzing ART outcomes based on gender and ethnicity

Preliminary results from the Artemis, GRACE and CASTLE studies were presented at AIDS 2008 where virologic, immunologic, and safety data from different ARV regimens were analyzed with respect to gender and ethnicity. All three studies used PI-boosted regimens as treatment modalities: Artemis compared darunavir/ritonavir to lopinavir/ritonavir in treatment-naïve patients at 48 weeks, GRACE evaluated lopinavir/ritonavir for safety and efficacy in treatment-experienced patients after 24 weeks of follow-up and the CASTLE study compared atazanavir/ritonavir with lopinavir/ritonavir in treatment-naïve patients at 48 weeks [25-28]. At baseline, patient characteristics were comparable across subgroups.

While the three studies varied in cohort size and the relative distribution of men versus women and ethnic representation, all three found no significant difference of clinical relevance in treatment outcomes (based on virologic suppression and CD4+ count), regardless of treatment modality, between men and women. There were also no significant differences in adverse events (most commonly nausea, diarrhea, rash, headaches and weight gain) when stratified and analyzed by gender, and the safety and efficacy profile for women in the GRACE study was comparable to other studies comparing PI-boosted regimens.

When comparing treatment efficacy and the incidence and frequency of adverse events among different ethnic groups, the Artemis study produced comparable results regardless of race. Ethnicity data from the CASTLE study indicated that while both treatment modalities in this study provided promising virologic and immunological results across ethnic sub-groups, there was some variation in the magnitude between different ethnic/racial groups, with ranges of those reaching undetectable levels of viremia ranging from 71% to 83% in the atazanari/r arm to 73% to 90% in the lopvinavir/r arm, with the lowest proportion of those achieving undetectable viremia among African Americans and the highest proportion among Asians or Caucasians. Similar immunological results were reported with respect to CD4+ count increases. There were modest differences in adverse events noted, with diarrhea higher among Caucasians (14%) than among African Americans (5%).

### Prevention research

Research into new HIV prevention technologies in the years leading up to AIDS 2008 has been discouraging. Between 2006 and 2008, five advanced stage trials (four for broad-spectrum microbicides and one for a vaccine) announced nil or negative results, and there are currently no vaccine candidates ready for field-testing. The long-term circumcision results reported at AIDS 2008 indicated that prevention interventions do have the potential to substantially reduce new infections. Other presentations mapped out prevention strategies using antiretroviral agents, which could be introduced in the field relatively quickly.

#### More evidence that circumcision works

**Figure 6:**
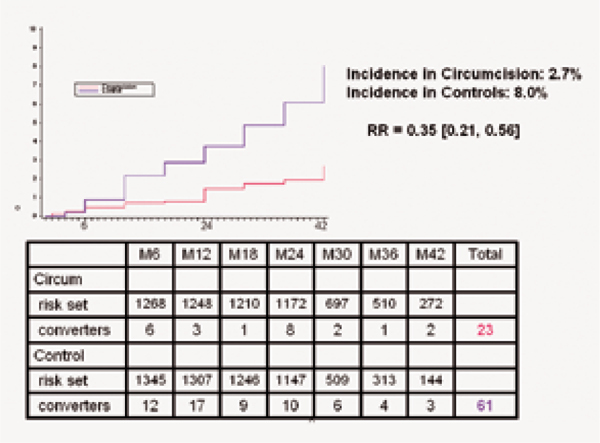
**Cumulative HIV seroincidence over 42 months by circumcision status**. Source: Bailey, RC et al. The protective effect of male circumcision is sustained for at least 42 months: results from the Kisumu, Kenya trial (THAC0501).

Most optimistically, Robert Bailey (Chicago School of Public Health, USA) reported results from extended follow-up of participants in their randomized controlled trial of male circumcision in Kisumu, Kenya at a late-breaker session. Previously, participants in the three trials of circumcision had data up to only 21 - 24 months post-randomization. Now, with the inclusion of 42 months of follow-up, Bailey and his colleagues reported a 65% protective effect of circumcision against HIV acquisition in young men in Kisumu (Figure 6). The Kisumu trial also attempted to gauge the effect of circumcision on sexual pleasure and performance. They found that there was no appreciable difference between circumcised and uncircumcised men in their reports concerning various measures of sexual function and satisfaction of female partners [29].

Bertran Auvert (Hôpitaux de Paris, Université de Versailles Saint-Quentin, France) presented the results of a study subsequent to his group's circumcision trial in Orange Farm, South Africa. After counselling 1,207 men on safe sex and treating them for sexually transmitted infections, the researchers offered free circumcisions in a medical clinic. Among the uncircumcised men (68% of the total), 65% eventually accepted the offer [30].

Auvert's group and the Kisumu trial also attempted to gauge the effect of circumcision on sexual pleasure and performance. They found that there was no appreciable difference between circumcised and uncircumcised men, according to reports from both males and females [31].

Fred Sawe (Kenya Medical Institute/Walter Reed Project HIV Program, Kenya) in addition reported that HIV prevention and reproductive health training was very well received during traditional male rites of passage in the Great Rift Valley [32]. These ceremonies include circumcision. In the interest of safety, medically trained personnel are increasingly invited to perform the actual circumcisions during maturation ceremonies.

Auvert presented the results of the first circumcision randomized clinical trial three years ago at the 3rd IAS Conference on HIV Pathogenesis and Treatment in Rio de Janeiro [33]. Studies began reporting a correlation between higher circumcision rates and lower HIV in parts of Africa 20 years ago [34]. WHO has since then developed a "Male Circumcision Quality Assurance Guide" providing a framework for safe mass circumcision programs, including infection control and risk reduction guidelines [35]. Yet there are still no large-scale circumcision programmes for high HIV prevalence areas. Richard White (London School of Hygiene and Tropical Medicine, United Kingdom) presented a model predicting that in sub- Saharan Africa, the lowest cost per HIV infection averted in the next ten years, around US$1,000, would occur if the target circumcision age group were 25-34 year-olds [36]. This is the male age range with the highest immediate HIV risk and somewhat older than the WHO recommended target group (12-30 year-old males). White and a poster presented by Agnes Binagwaho (National AIDS Control Commission, Rwanda) advocated also circumcising newborns, although the benefits of doing so would not be visible for approximately 25 years [37].

#### Harnessing antiretroviral treatment for prevention

While the implementation of circumcision as a viable prevention strategy lags, a debate has emerged about using antiretroviral therapy as a prevention tool. In January 2008, Switzerland's Federal Commission on AIDS-Related Issues (EKAF) released a statement indicating that people living with HIV who were taking an effective (maximally suppressive) antiretroviral regimen could not transmit the virus [38,39]. There were several strict conditions attached to that position, including at least six months of undetectable viral load, no other sexually transmitted disease, and ensuring HIV-negative sex partners were able to make an informed choice to dispense with condoms.

Nonetheless, arguments ensued over the Swiss statement's implications at an EKAF-sponsored satellite symposium that took place just before the official opening of AIDS 2008. EKAF president Pietro Vernazza presented his Commission's point of view, which places the position in a very carefully-defined context [40]. He said that EKAF was merely standardizing what physicians were already telling their patients. In addition, Swiss law is very strict about exposing other people to HIV, even if transmission does not occur. Vernazza's point was not that the risk of transmission under suppressive therapy was nil, rather that it was very small, comparable with the risk of transmitting while using condoms. Successful ART should therefore be a reasonable defence against laws criminalizing HIV exposure.

Suzanna Attia (University of Bern, Institute of Social and Preventive Medicine, Switzerland) presented the results of a meta-analysis of studies investigating transmission risk under ART [41]. There have been no studies of patients on successful ART or in men who have sex with men. There have been a few studies concerning HIV-discordant heterosexual couples in which the HIV-positive partner has an untreated viral load below 400 copies/mL. One transmission was noted in a total of approximately 900 patient-years. Information on condom use and sexually transmitted infections in these studies was unavailable. Attia argued for further research before drawing any conclusion.

A randomised controlled trial is currently under way to quantify the relationship between the level of treatment-suppressed viral load and HIV transmission. The trial, sponsored by the US National Institutes of Health, will follow 1,750 HIV sero-discordant couples assigned to immediate or deferred treatment. Results will not be available before 2016, a problematic timeline given the research suggesting that persons with treatment-suppressed HIV are already reducing their condom use [42].

#### The tantalizing promise of PrEP

In addition to viewing ART as a potentially effective prevention modality involving those already infected, at the conference there was also discussion of administering ART as pre-exposure prophylaxis (PrEP) for HIV-negative persons who are at high risk for infection, including members of HIV-discordant couples [43]. Although it faced some activist opposition in the first round of Phase III clinical trials, when some trials were prematurely halted, support for the concept has grown, no doubt at least partly due to the failure of several other biomedical prevention modalities and improvements in the engagement of civil society in the design and implementation of PrEP clinical trial protocols.

**Figure 7:**
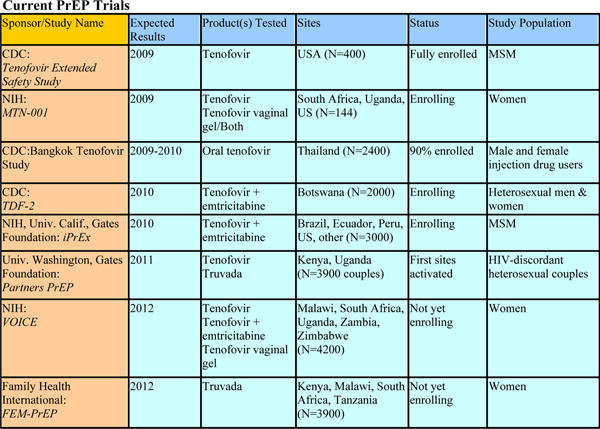
**Current PrEP trials**. Source: Mastro, T, Pre-exposure prophylaxis. Overview of current and planned trials (THSY0603).

There are a number of on-going and planned PrEP trials testing daily regimens (Figure 7). Although PrEP was first discussed in the mid 1990s, it will be at least four more years before we see PrEP's benefits fully evaluated in several populations using multiple dosing strategies. The utility of intermittent precoital regimens is only now coming under consideration [44,45]. There was one preliminary human trial reported at AIDS 2008 involving long-term injectable PrEP. This trial found than an intramuscular sustained-release form of rilpivirine (TMC278, a new reverse transcriptase inhibitor nearing licensure for HIV treatment) could deliver protective drug levels for over 12 weeks.

There are obvious safety and economic concerns about supplying antiretroviral drugs to large HIV-negative populations. If it proves effective, topical application to genital and anal areas might be more feasible. As conference attendees heard, tenofovir gel applied locally yields vaginal drug concentrations 100- to 1,000-fold higher than systemic oral administration [46]. In contrast, blood plasma concentrations are more than 10-fold lower with the gel.

Topical microbicides have not performed well in human HIV prevention studies, with 10 trials of surfactant and polyanionic compounds yielding negative results. These non-specific, broad-spectrum compounds inactivate bacteria, viruses and even sperm by emulsifying (surfactants) or coating (polyanions) their outer layers.

The field is clearly moving forward. AIDS 2008 marked the shift from broad-spectrum to antiretroviral microbicides [47,48]. Besides the VOICE study testing oral PrEP versus tenofovir gel, another large, advanced-stage trial will start in the next two years. This is IPM009, still in its planning phase. It will study the efficacy of the reverse transcriptase inhibitor dapivirine, either as a short-acting vaginal gel or as a long-lasting vaginal ring. The latter is a new, innovative mechanism for delivering anti-HIV microbicides. IPM009's sponsor, the International Partnership for Microbicides, eventually plans to combine dapivirine with the licensed entry inhibitor maraviroc.

But the results of many of these trials are not expected until 2010-12. The only encouraging microbicide *in vivo *results at AIDS 2008 came from a small macaque trial of a vaginal gel, combining two antiretrovirals, tenofovir and emtricitabine. The trial applied this combination gel 30 minutes before exposure to HIV. Six out of six macaques were protected from 20 vaginal challenges over ten weeks. In contrast, seven of eight control macaques became infected after a median of 3.5 challenges [49].

#### Vaccines: the role of protective immunity

Recent setbacks in both the vaccine and microbicides field have required a refocus of research efforts in both fields. At the conference, Seth Berkley (International AIDS Vaccine Initiative, USA) reviewed the International AIDS Vaccine Initiative's (IAVI) efforts to screen human subjects for broadly neutralizing antibodies against HIV [50]. These antibodies would prevent new cells from becoming infected and might be the key to a vaccine that creates "sterilizing immunity" against a wide variety of HIV isolates. Berkley says that advanced mass screening techniques have now permitted IAVI's antibody project to select promising candidates. Even if such antibodies were isolated in a few individuals, the problem would remain how to induce them in the entire human population. A vaccine based on these antibodies is not expected soon. Berkley also promoted the concept of using replicating viral vectors. These vectors would consist of a carrier virus containing recombinant HIV genes that would induce a potent immune response.

Current HIV vaccine vectors are all nonreplicating. Among these is the adenovirus vector used in the Merck vaccine that yielded negative results in the well-known STEP trial terminated last year. A US National Institutes of Health study comparing chimpanzee responses to replicating and nonreplicating adenovirus-based HIV vaccines was one of the early warnings about the Merck vaccine design [51]. The replicating adenovirus vector elicited markedly superior immune responses. Switching to live vectors now will entail considerable delay as various technical and safety concerns are resolved.

#### A new emphasis on prevention cocktails or "combination prevention"

Combination prevention strategies was a popular topic of discussion at the conference [52]. Circumcision programmes can be combined with condom promotion and other structural and socio-behavioural approaches to preventing HIV. More detail on combination prevention is outlined in the following section.

As the Kisumu trial results show, circumcision alone will not eradicate HIV incidence in men. There is some optimism that adding antiretroviral agents in one of the forms described above may have a major impact in reducing incidence. Research is moving forward slowly despite this potential, and mass implementation will likely also be slow, if the circumcision experience is any example. The prevention research legacy of AIDS 2008 is an increasing recognition that fulfilling the promise of emerging prevention technologies requires a renewed sense of urgency.

## Conclusion

AIDS 2008 became known for the "marriage" of treatment and prevention, with many speakers underscoring the need to integrate prevention and treatment interventions to ensure a more effective and sustainable response to HIV/AIDS. The term 'combination prevention' - the need to integrate a full range of biomedical, socio-behavioural and structural interventions is covered in greater detail in Section 5 of this supplement. New advances in the clinical management of HIV in resource limited settings and the success of approaches such as task-shifting could serve as important models for ART expansion as the HIV field enters what is increasingly known as the 'second wave' of ART access. However, cautions regarding the potential emergence of resistance strains and pressures on health care systems are highlighting the need for operations research to ensure the optimal management in treatment delivery, including ensuring low-cost second line regimens are available.

The vaccine field is returning to the laboratory to assess different options, such as harnessing the body's innate immunity using non-replicating viral vectors. But the only two recently proven or promising biomedical interventions at this point are circumcision and ART-based prevention, either as PrEP or as part of increased ART scale-up that - it is hypothesized - will have a significant impact on community load and the course of the epidemic.

## Competing interests

Mark Mascolini, Rodney Kort and David Gilden are independent consultants contracted by the International AIDS Society for the purpose of preparing and editing the AIDS 2008 Impact Report for publication.

## Authors' contributions

Mark Mascolini and David Gilden drafted the initial text for this section and Rodney Kort provided editorial input and advice. All authors have approved the manuscript for publication.
